# An Intervention to Enhance Social, Emotional, and Identity Learning for Very Young Adolescents and Support Gender Equity: Protocol for a Pragmatic Randomized Controlled Trial

**DOI:** 10.2196/23071

**Published:** 2020-12-31

**Authors:** Megan Cherewick, Sarah Lebu, Christine Su, Ronald E Dahl

**Affiliations:** 1 Department of Health Sciences California State University East Bay Hayward, CA United States; 2 Institute of Human Development University of California Berkeley Berkeley, CA United States

**Keywords:** developmental science, adolescence, adolescence interventions, gender norms, developmental evaluation, gender, social learning, emotional learning, identity learning, adolescents

## Abstract

**Background:**

The onset of puberty is a pivotal period of human development that is associated with significant changes in cognitive, social, emotional, psychological, and behavioral processes that shape identity formation. Very early adolescence provides a critical opportunity to shape identity formation around gender norms, attitudes, and beliefs before inequitable gender norms are amplified during and after puberty.

**Objective:**

The aim of the Discover Learning Project is to integrate strategic insights from developmental science to promote positive transformation in social, emotional, and gender identity learning among 10- to 11-year-olds in Tanzania. Through a pragmatic randomized controlled trial, the intervention scaffolds the development of critical social and emotional mindsets and skills (curiosity, generosity, persistence, purpose, growth mindset, and teamwork) delivered by conducting 18 after-school, technology-driven, experiential learning sessions in small, mixed-gender groups.

**Methods:**

The Discover Learning Intervention is a 3-arm randomized controlled trial that will be delivered to 579 participants selected from four public primary schools in Temeke District, Dar es Salaam, Tanzania. Randomization will be done at the individual level into 3 treatment groups receiving incremental intervention components. The treatment components include Discover Learning content curated into child-friendly videos, facilitated discussions, and a parent-child workbook, to be implemented over two phases, each 6 weeks long. A baseline survey will be administered to participants and their parents prior to the intervention. The process will be observed systematically, and data will be collected using surveys, in-depth interviews, observations, and focus group discussions with adolescents, parents, teachers, and facilitators conducted prior, during, and after each implementation phase.

**Results:**

This study builds on formative and pilot studies conducted with the target population to inform the design of the intervention. The results will generate new evidence that will inform strategies for achieving scale in Tanzania and provide insights for replication of similar programs that are invested in gender-transformative interventions in peri-urban, low-resource settings.

**Conclusions:**

The Discover Learning Intervention makes an important contribution to the field of adolescent developmental science as an intervention designed for very young adolescents in a low-resource setting.

**Trial Registration:**

ClinicalTrials.gov NCT04458077; https://clinicaltrials.gov/ct2/show/NCT04458077

**International Registered Report Identifier (IRRID):**

DERR1-10.2196/23071

## Introduction

### Overview

The period of developmental changes and rapid growth during adolescence presents a key opportunity to promote positive lifetime health and well-being trajectories that have enduring impact throughout life [1]. Very early adolescence, before the onset of puberty (10-11 years old), is a pivotal transition from childhood into adulthood that can benefit from developmentally informed programming and strategic investment. Changes during this period include rapid physical growth and brain development, sexual maturation, and changes in cognitive, social, emotional, psychological, and behavioral processes. The period of brain development from the onset of puberty may represent a unique combination of stability and plasticity in developing neural networks that facilitate learning and experience [2]. During this distinctive maturational window, adolescents are particularly sensitive to learning opportunities that can shape social, emotional, and identity development [3]. A growing body of evidence indicates that adolescents between the ages of 10 and 14 actively build their identities, establish behaviors, gain social knowledge, and shape their values and beliefs during these years [1,2,4]. Furthermore, very early adolescence is an opportune window to shape identity formation around gender norms, attitudes, and beliefs before inequitable gender norms are amplified during and after puberty.

Brain development during early adolescence is naturally aimed at discovery learning in the social, emotional, and identity learning domains. The onset of puberty is associated with two important maturational changes that impact learning: (1) an increase in the tendency to explore, discover, and seek novelty/excitement; and (2) an increase in natural curiosity to explore and understand one’s social world, including social roles, social hierarchies, issues of social acceptance, admiration, and learning to establish individual identity [5]. Early learning experiences during adolescence shape identities in ways that have profound implications for health—especially sexual and reproductive health and vulnerability to gender-based exploitation*.*

The Lancet Commission on Adolescent Health and Wellbeing report highlighted the need for investment in the largest generation of 10- to 24-year-olds in human history [6]. By 2018, there were approximately 1.24 billion adolescents, representing 16% of the global population [7]. Research indicates that adolescence is a period of vulnerability where physical and mental health problems emerge, which can persist into adulthood [8]. Increases in accidents, suicides, homicide, mental disorders, substance use, eating disorders, sexually transmitted diseases, and unintended pregnancy can lead to lifelong negative trajectories. While interventions have worked to address these risks, they often target older adolescents (aged 15-19 years) and have limited impact on very young adolescents [9,10].

### Adolescence in Tanzania

In low-resource contexts, adolescents face additional stress and adverse life experiences. As a result, there has been an increasing focus on the need to target early adolescence to improve health trajectories for sub-Saharan African youth [11]. Evidence suggests that interventions that target very young adolescents can have sustained impacts such as decreasing the spread of HIV/AIDS, decreasing the number of unwanted pregnancies, and improving health and well-being [12-14]. Tanzania is experiencing a surge in the youth population, with about half the population younger than 17.5 years and 47% younger than 15 years [15,16]. The population in those age groups is expected to double by 2055 [7]. The Global Out-Of-School Children Study estimated that approximately 3.5 million school-aged children and adolescents were not in school in 2017 [17], and the results of a child poverty study released in 2016 showed that 74% of children in Tanzania are affected by multidimensional poverty while 29% live in households below the monetary poverty line [18].

Since the introduction of free primary education in 2001, Tanzania has made strides to improve access to education. Between 2004 and 2010, enrolment in secondary education tripled for girls and quadrupled for boys, and by 2011 over 94% of children aged 7 to 13 years were enrolled in school [19]. While the introduction of free primary education has resulted in higher secondary education enrolment, it has also revealed important equity gaps. Only a third of children that start primary school complete the cycle in seven years. The transition rates largely favor boys, with approximately 21% of boys joining secondary schools compared to 16% of girls [20].

In 2016, 1 in 4 adolescent girls aged 15-19 years had begun childbearing, reflecting a 4% increase in teenage pregnancy since 2010 [21]. Unfavorable sexual and reproductive health outcomes for girls have been attributed to the fact that Tanzanian youth face highly contrasting norms around gender and adolescent sexuality. Gender roles are differentiated, with boys allowed more freedom outside the house, whereas girls are perceived to be better suited for home chores [22,23]. A cultural prototype of a chaste female student is highly valued. At the same time, female sexuality is perceived as a resource intended for exploitation, and transactional sex is often considered a young woman’s sole commodity. Disparities in enrolment patterns and educational outcomes have led researchers to focus on the role of gender and sexual and reproductive health in education.

Investing in programs for very young adolescents can help address gender inequities through inclusion of gender-transformative content that includes mixed-gender learning in social groups before puberty and sexual debut. Previous studies targeting the sexual and reproductive health of adolescents in Tanzania often focused on later adolescence (ages 15-19 years) and missed the opportunity to shape social, emotional, and identity learning during early adolescence, which can be transformative during later adolescence. More research is needed to understand what impact investment in very early adolescence can have on improving multiple health outcomes as well as transforming gender behaviors, attitudes, and beliefs.

### Technology as a Learning Tool

Advances in technology provide an increasingly important social learning context and access to information and new learning opportunities. By 2017, use of mobile phones in Tanzania was at 80% [24]. The changing technological landscape in low-income countries is a nascent opportunity to advance learning and address inequities in overburdened, underresourced education systems [25]. In Tanzania, one program that has integrated the use of technology in learning is the BridgeIT project, which reaches 80,000 pupils across 150 primary schools [26,27]. Adolescents are often early adopters and are motivated to learn using new technology, particularly those that enable them to gain social support [28]. The natural motivation of adolescents to explore, discover, and master novel and stimulating environments is an opportunity to deliver a high-impact intervention through technological platforms. Small advances have been made toward leveraging technology as a tool for promoting positive social and emotional skills and mindsets among adolescents. Examples of such programs have demonstrated positive impact on mental health and have reduced bullying among adolescents [29,30].

This study explores the design of a social and emotional learning intervention to promote gender equity among very young adolescents. The study hypothesis is that targeting a window of opportunity for very young adolescents (aged 10-11 years) and supporting development of social and emotional mindsets and skills through experiential learning in small, mixed-gender groups will promote social and emotional learning and identity development that has a positive impact on gender equity and associated health outcomes [3]. To test this theory of change, we measure social and emotional mindsets and skills and measures of gender norms, attitudes, and beliefs to capture the positive impact of the intervention on these outcomes. Other objectives of this study are (1) to test the effectiveness of providing learning opportunities that focus on specific social and emotional skills and mindsets (including curiosity, generosity, persistence, purpose, growth mindset and teamwork and gender equity); (2) to evaluate the use of digital technology for social, emotional, and identity learning; and (3) to identify aspects of high-impact learning opportunities with the potential to be scaled in low-resource settings across Tanzania and in similar low-resource contexts.

## Methods

### Study Location

*Discover* will be conducted in Temeke Municipality in Dar es Salaam, Tanzania. Temeke District is the largest of Dar es Salaam’s three districts. It is unique since it encompasses both metropolitan urban and rural areas. It has a sociodemographic mix of people from all parts of the country. There are 114 primary schools in Temeke with more than 130 pupils per classroom on average. The number of primary school–going children in the municipality is 170,477. Of these, 84,371 are boys and 86,106 are girls [31].

### Overview of Discover Learning Project

The primary aim of the Discover Learning Project (*Discover*) is to test an intervention for very young adolescents to promote positive social and emotional skills and mindsets that have the potential to transform gender norms and attitudes. A secondary aim is to better identify effective components of *Discover* that are scalable and require the fewest resources to implement. The study aims are detailed in Textbox 1.

***Discover* study design.**

**Research aims:**

Compared to matched controls, do participants in *Discover* show the following?

- Decreased experience of gender inequality
- More positive social relationships with peers and trusted adults
- Enhanced feelings of empowerment and motivation to engage in school and other learning experiences
- More positive attitudes toward and comfort with using technology as a learning tool

A secondary aim is to identify high-impact components for scale at low cost.

**Selection of study sites:**

- The profile of the schools should be representative of an average school found in Tanzania in terms of socioeconomic status, religion, amenities, etc.
- Schools should be a public day school where a majority of pupils reside within a walking distance from the school.
- Schools should not have had an existing behavioral intervention within the last 7 years targeting the target age group.
- Schools should have a large enough number of 10- to 11-year-olds to obtain sample size required for study.

**Participant eligibility criteria:**

- Must be a 10- to 11-year-old student in Grade 3, 4, or 5 in any of the 4 selected study sites
- Must have agreed to participate in the study
- Parent must consent to the study and provide written parental permission

The project draws on developmentally informed principles that balance autonomy with adult engagement to scaffold social and emotional learning [32]. The specific target areas of adaptive social and emotional mindsets and skills include growth mindset, curiosity, generosity, persistence, purpose, prosocial behavior, and teamwork. The study is implemented in small mixed-gender groups by introducing positive, socially scaffolded exploration and use of digital technology. *Discover* integrates insights from developmental science that are matched to developmental changes during early adolescence in three ways:

1. By focusing on social learning in mixed-gender groups.
  Adolescents have a natural motivation for social status, prestige, and respect. Mixed-gender groups provide opportunities for positive, collaborative, and scaffolded learning that integrate content that recognizes girls and boys as equal in solving problems.
2. By building upon a core concept of discovery learning.
  Adolescent-driven discovery learning creates opportunities for positive risk-taking that results in healthy, positive, productive, high-arousal learning.
3. By using technology to deliver learning experiences.
  Technology takes advantage of adolescents’ increased tendency to seek novelty, excitement, and mastery and helps them adapt successfully to the increasing influence of technology in the world around them. 

The content and activities in *Discover* are focused on enhancing and supporting skills and mindsets that motivate adolescents to feel respected and admired by the adults and peers in their lives [3]. The adaptive areas of social and emotional mindsets and skills include *positive gender norms*—encouraging positive gender norms and exploration of gender identity can disrupt inequitable gender norms and support gender equity; *teamwork*—activities geared toward teamwork can promote positive peer interactions, encourage communication skills, and provide appreciation to and from peers; *growth mindset*—focusing on the potential to develop personal abilities; *curiosity*—curiosity and experimentation are highly arousing and motivate learning; *purpose*—exploring one’s purpose can help develop long-term, heartfelt goals; *persistence*—focusing on ways of increasing coping skills for rapid physical, social, and emotional changes that are associated with early adolescence; and *generosity*—encouraging intentional practice of gratitude and kindness in order to foster better interpersonal skills.

Each of these mindsets and skills have been adapted into scripted videos that are culturally sensitive and easy to understand for 10- to 11-year-olds. In addition to watching the videos, the adolescents will be immersed into a series of experiential and interactive learning activities specific to each mindset and skill. A detailed description of each social and emotional mindset and skill and the associated intervention activities can be found in Multimedia Appendix 1.

### Intervention Components

The intervention design focuses primarily on increasing positive gender norms and boosting the outcome of each social and emotional mindset and skill among study participants. The intervention includes the following elements.

#### Youth Sessions

1. Ubongo Kids videos: Students will watch engaging digital learning content developed for children in East Africa by Ubongo Kids.
2. Team building: Students will be placed in small mixed-gender teams for self-guided discussions and activities that are designed to facilitate teamwork and provoke discussions on gender.
3. Reflection: At the end of each session, students will be encouraged to reflect about what they learned during the session as a team as well as reflect on how this might apply to their life.
4. Technology 101: Students will be introduced to the basic components of a tablet and taught how to turn them on and off as well as how to navigate to files and programs.
5. Tablet games: Students will work in pairs on simple games designed with increased difficulty. The games the youth play will be modified versions of Tic-Tac-Toe, Pong, and a mathematical/numeracy game.
6. Community mapping: Students will be taken around their community to help them identify different places in the community that contain resources of importance.
7. Mind mapping: This mapping activity will look at important aspects in a student’s life and help them consider their identities and roles as individuals, in their family, and in the community.
8. Kanga project: Youth will be encouraged to think about a value that they or their community have and represent this in a fabric commonly used in East Africa.
9. Parent-child workbook: The workbook will have class activities that youth fill out during the sessions, as well as home activities, which will be simple questions or activities that reflect what they will have learned during the sessions.
10. Community event: At the end of the project, students will present their kanga artwork and gifts in an event that will bring together children and caregivers, members of the local government and ministries, and teachers.

#### Parent and Caregiver Sessions

For each implementation phase, *Discover* will hold three one-hour sessions every other week over 6 weeks with parents and caregivers. Structured parent sessions are an opportunity for caregivers to ask questions about the intervention or the parent-child workbook. The parent-child workbook is designed to reinforce learning within the home by providing discussion questions and short activities. A community event will be held at the end of each intervention phase that brings together the participant youth, parents and caregivers, members of the community advisory board, members of the local government, and teachers. Students will get a chance to demonstrate the skills they will have learned during the intervention, showcase their kanga (traditional African fabric commonly designed with colorful patterns and messaging), and offer them as gifts to the community.

### Research Team Training

Core research team members will complete training on research ethics prior to the study. Their training will include confidential handling of data, obtaining consent and assent from study participants, and data collection and management. The field research team will be composed of 2 master trainers and 12 community facilitators. Training will be conducted in two phases. First, the master trainers will be trained over two weeks by the *Discover* Project Manager. The training will cover research ethics, gender-transformative content, technology use, and partnering with the community. Second, community facilitators will be trained with support from the master trainers over eight days. During the training, 6 facilitation principles will be adapted to facilitate discovery learning. They are (1) scaffolding learning to create a safe space for learners, (2) emphasis on learning over education, (3) withholding judgment since learning is a nonlinear process, (4) encouraging teamwork and positive group dynamics, (5) disrupting gender norms, and (6) encouraging a growth mindset. Sessions will use a combination of presentations, discussion, practice, and reflection.

### Participant Recruitment and Eligibility Criteria

**Figure 1:**
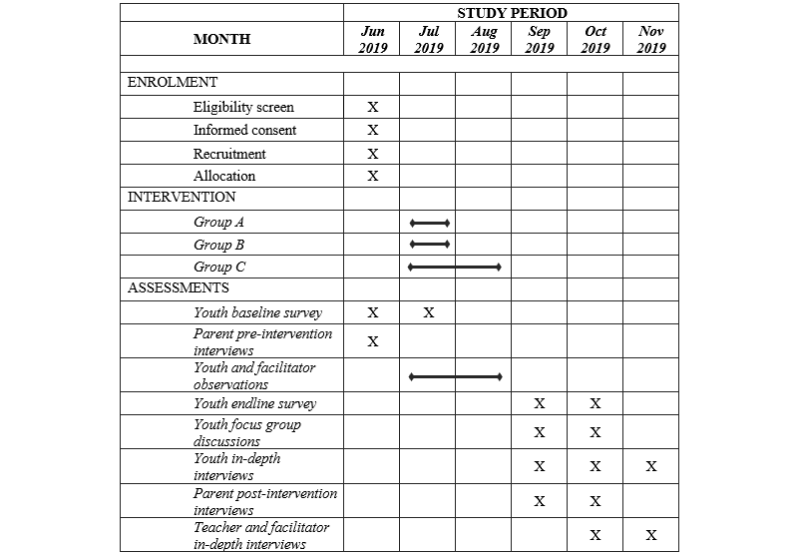
Study recruitment and implementation schedule.

Participants will be recruited from four primary schools from low-income urban neighborhoods of Temeke District. They will be selected based on the following criteria: (1) the school should not have had an intervention within the last 7 years that included a behavior change component, a teachers’ capacity-building component on soft skills (eg, positive discipline or facilitation skills), or a gender equity program; (2) the school should be a public/government school; (3) the school should have a large enough number of early adolescents to obtain the sample size required for study; and (4) the school should be representative of an average school in Tanzania in terms of socioeconomic status, religion, amenities, etc. Participants will be eligible if they meet the following criteria: (1) they must be a 10- to 11-year-old student in Grade 3, 4 or 5 in the 4 selected study sites; (2) they must verbally assent to participate in the study; and (3) their caregiver must provide consent for them to participate in the study. The after-school intervention will be conducted with a total of 579 youth. Students will be randomized into Groups A, B, and C. Figure 1 provides an overview of the scheduling of the intervention and research elements. A complete checklist listing the items to address in a trial protocol according to the CONSORT (Consolidated Standards of Reporting Trials) guidelines can be found in Multimedia Appendix 2 [33].

### Randomization Procedure

Participating 10- to 11-year-olds from grades 3, 4, and 5 will be identified from each school. At each school, we will hold an event where we line up eligible youth outside their classrooms. A research assistant will hold a box with pencil sharpeners of different colors inside. The box will have a small hole large enough to fit a hand through but small enough that students cannot see the pencil sharpeners. Each youth will select one sharpener at random. Students will be assigned to groups A, B, or C depending on whether they pick pink, yellow, or blue pencil sharpeners, respectively. Youth will then be matched to their selected pencil sharpener with a research assistant holding the same color, and that research assistant will register students to the assigned group. Table 1 lists the expected results of randomization to the three groups, according to the sample size calculation, and the intervention components that will be administered to each group.

**Table 1:** Intervention components offered per study arm.

Study components	Group A (n=186)	Group B (n=185)	Group C (n=208)
Number of sessions	6	6	18
Ubongo Kids videos	✓	✓	✓
Mixed gender groups of size	15-26	4-5	4-5
Discussion and activities	✗	Self-guided	Guided by trained facilitators
Parent-child workbook	✗	✗	✓

### Study Instruments

A mixed-method approach will be used to capture the innovation process of project implementation. Qualitative methods will be sought to capture perspectives from youth participants, parents and caregivers, and community members. Qualitative tools to be used include in-depth interviews, focus group discussions, and participant and facilitator observations. The mixed-method evaluation design is detailed in Textbox 2.

**Data collection methods.**

**QUALITATIVE METHODS**

**Adolescent**

- In-depth interviews
- Focus group discussions
- Session materials
- Parent-youth workbook

**Parent**

- In-depth interviews (pre- & post-intervention)
- Parent session materials

**Facilitator**

- Session debrief reflections
- Challenges, solutions, and adaptation logs
- In-depth interviews

**Participant and facilitator observations**

**QUANTITATIVE METHODS**

**Adolescent Survey**

- Sociodemographic information
- Social and emotional mindsets and skills
- Discrete choice experiment
- Parent-youth workbook

#### Youth Surveys

The evaluation surveys for youth in *Discover* have been developed following a review of existing, validated tools that have been previously used in similar low-resource settings and tested through multiple pilot field tests with young adolescents in study sites. The surveys have been adapted through an iterative process following the pilot intervention and through meetings with Tanzanian and US research evaluation team members. Some of the measures that have been adapted include the following: gender equality—Gender Roles, Equality and Transformations [34]; curiosity—the Trait State Curiosity Scale [35]; empathy—the Empathy Questionnaire for Children and Adolescents [36]; growth mindset—Dweck Growth Mindset Questions [37]; and technology use and uptake—Media and Technology Usage and Attitudes Scale [38].

Final survey measures have been reviewed for relevance, cultural meaning, and acceptability by youth. Following this, the survey will be transferred to a tablet-based questionnaire for use in one-on-one interviews. The survey will be translated and back-translated from English to Swahili. The final *Discover* measures are listed in Multimedia Appendix 3.

#### Discrete Choice Experiment

We have developed a discrete choice experiment to assess adolescents’ gender perceptions, attitudes, and roles. This is a quantitative technique for eliciting individual preferences and hence informing policy, planning, and resource allocation decisions [39]. Typically, in a discrete choice experiment, study participants are repeatedly presented with scenarios on several attributes and asked to state their preference. In our approach, adolescents will be presented with 3-5 scenarios under each attribute and asked to decide whether the scenarios best describe boys, girls, or both boys and girls. The attributes will complement the social and emotional mindsets and skills from our intervention modules. Response options will be presented as cartoon images of boys only, girls only, or both girls and boys; participants will have to pick one image for each scenario.

#### Qualitative Data Collection

In-depth interviews with youth and parents will be conducted at the start of the program over a period of 6 weeks. Interviews with youth will be conducted by trained qualitative researchers after school hours. Parent interviews will be conducted over the phone at a time preferred by the parent. During classroom sessions, trained observers will keep notes of youth and facilitator engagements. This will be done throughout the project. Upon completion of the project, endline in-depth interviews with youth and parents lasting approximately 30 minutes will be conducted. In-depth interviews will be conducted with all facilitators. Further, focus group discussions will be held with groups of 4-6 youth, and in-depth interviews with teachers will be completed. Recordings of all interviews and focus groups will be transcribed in Swahili and then translated to English by project staff.

### Sample Size and Power

The sample size of 579 was chosen to be able to measure the minimum detectable effect of the *Discover* intervention on outcomes of social and emotional mindsets and skills. A sample of 186 students per study group produces an effect size of 0.28 and 80% power, assuming a 1-sided *t* test and α level of .05 to allow for multiple testing across groups. Randomization will be done at the individual level because the research protocol poses minimal risk for contamination. The project does not have a prior estimate of the intracluster correlation coefficient between individuals nested within a school, so we have used 0.39, adapted from similar studies on social and emotional interventions in sub-Saharan Africa [40]. The control groups (A and B) will each have 186 students, and the remaining participants will be in the treatment group (Group C).

### Data Analysis

Quantitative statistical data analysis will be conducted using Stata SE 16.0 (StataCorp). For each group, we will test using an intent-to-treat model to determine the effectiveness of each group on (1) social and emotional mindsets and skills outcomes (curiosity, generosity, persistence, purpose, growth mindset, and prosocial behavior) and (2) gender norms, attitudes, and beliefs. Descriptive statistics will be calculated for each measure per intervention group and will be used to check for skewness and data non-normality. The psychometric properties of each measure will be assessed using confirmatory factor analysis to assess the adequacy of factor structures suggested by previous studies. Additional analyses will be conducted using *t* tests and logistic regressions to explore potential confounders of these relationships such as age, gender, and assigned facilitator. Validated measures will be used for structural equation modelling to test the relationships between intervention groups on social, emotional, and identity learning measures. For each model tested, structural equation modelling will be used to test (1) overall fit, (2) the significance of structural paths, and (3) the amount of variability of the latent variables accounted for by observed variables. Model fit will be assessed by using goodness-of-fit indices including the chi-square, the root mean square error of approximation [41], the comparative fit index [42], the Tucker-Lewis index [43], and the standardized root mean residual.

Qualitative data analysis will be conducted in ATLAS.ti (ATLAS.ti Scientific Software Development GmbH). After completion of interviews and focus groups, a Tanzanian translator will complete translation from Swahili to English. The translations will be cross-checked by researchers in Tanzania. Transcripts will be coded using grounded theory methodology, and content analysis will be performed to identify key themes. Quotes will be selected as exemplars of these themes. Additional qualitative materials include participant observations, facilitator debrief reflections, parent session notes, and teacher interviews. Each of these documents will be translated from Swahili to English by the research team and summarized, and key themes will be extracted. Artifacts from youth will be collected throughout the intervention implementation to serve as exemplars of curriculum implementation.

### Availability of Data and Materials

The data sets that will be generated or analyzed during this study will not be made publicly available due to the sensitive age of the study participants (10- to 11-year-olds) at baseline but may be available from the corresponding author on reasonable request. The author will vet requests to be certain that appropriate institutional review board (IRB) approvals and data safety guidelines are in place before distribution.

## Results

This project was funded in November 2016 by the Bill and Melinda Gates Foundation. The University of California, Berkeley Committee for Protection of Human Subjects IRB approved this study (CPHS Protocol Number: 2017-01-9464; date: July 11, 2019). The primary local partner, Health for a Prosperous Nation, obtained ethical clearance for these research activities from the National Institute of Medical Research, the local IRB in Tanzania (Ref. NIMR/HQ/R.8a/Vol. IX/ 2491; date: May 15, 2019). In addition, the project sought and secured support from all local partners, Temeke Municipal Council and the Ministry of Education in Tanzania. Screening and enrolment of participants was done in June 2019. Data for the baseline survey was collected in July 2019.

The first phase of the intervention was delivered starting in the month of July 2019 for a period of 6 weeks to 579 participating 10- to 11-year-olds. Throughout the course of the intervention, systematic observations were carried out. Follow-up data was collected in the months of October and November 2019. The first phase of the intervention closed out in November 2019. At the time of writing this paper, data analysis had not yet been concluded. The second phase of the intervention kicked off with enrolment of participants in July 2020. This phase of the study targets the same participants from phase I and expects to reach 500 participants. The baseline survey will be conducted from August to September 2020. The intervention will be delivered remotely via technology for ten weeks from September 2019. The key findings from this phase of the project and the longitudinal data collected will be submitted for publication in peer-reviewed literature and presented at national and international conferences.

## Discussion

Findings from *Discover* will provide evidence of the impact of the different components of a developmentally informed social, emotional, and identity learning intervention for very young adolescents by comparing outcomes in groups A, B, and C and emergent themes from interviews and focus groups. Synthesis of quantitative and qualitative results will allow for identification of the highest impact components and resources required for the intervention. Results from structural equation modelling will be used to create a testable model of the relationships between social, emotional, and identity development on gender equity outcomes. These results will be used to develop a scalable, low-resource intervention program. This study tests the translation of developmental science principles to youth programs. In addition, findings have the potential to be replicated in other low-resource contexts. This study has some limitations. Randomization of adolescents will be done at the individual level and not the school level to maximize participation. Findings from the intervention will therefore apply to the individual level but cannot be extrapolated to the community level. Despite efforts to ensure intervention groups receive the package of components pertinent to that group, there is a possibility that some adolescents will discuss their group’s activities with friends and siblings, and therefore groups may become aware of intervention components received by other groups. The findings from this study will contribute to the evidence base on development science for very young adolescents. Further, the study design, methods, and evaluation will be useful for other studies that are invested in gender-transformative interventions through support of social and emotional learning. *Discover* will identify the impact of different intervention components that can be leveraged to replicate and scale up similar programs in peri-urban, low-resource settings.

## Appendix

Multimedia Appendix 1 Social emotional mindsets and skills targeted by Discover.

Multimedia Appendix 2 CONSORT Checklist.

Multimedia Appendix 3 Measurement scales used in Discover.

## Abbreviations

CONSORT: Consolidated Standards of Reporting Trials
IRB: institutional review board
