# Supplementary material for: An Intervention to Enhance Social, Emotional, and Identity Learning for Very Young Adolescents and Support Gender Equity: Protocol for a Pragmatic Randomized Controlled Trial
Source: JMIR Res Protoc. 2020 Dec 31;9(12):e23071. doi: 10.2196/23071 (PMC7808886; doi:10.2196/23071)
Supplement: Multimedia Appendix 1 [file resprot_v9i12e23071_app1.pdf]

Social emotional mindset and skill	Rationale and examples of activities
**Positive gender norms**	Gender identity is shaped by gender attitudes, beliefs and behaviors during early adolescence. Encouraging positive gender norms and exploration of gender identity can disrupt inequitable gender norms and support gender equality. *Discover* supports positive gender norm development through:<br>• Use of small, mixed gender group work with scaffolded facilitator support.<br>• Having participants reflect on how gender might influence group processes and how gender equality in groups can be an asset.<br>• Using pause and reflect moments, 'teachable moments', to include gender transformative reflection when boys and girls revert to traditional gender roles during an activity
**Teamwork**	Adolescents are sensitive to peer assessment, admiration and social status. Teamwork can promote positive peer interactions, encourage communication skills, provide opportunities to self-regulate, reward and appreciation to/from peers, and identifying one's own strengths and contributions. *Discover* supports positive teamwork through:<br>• Activities that employ different types of communication (verbal and nonverbal) is practiced and encouraged<br>• Reflection on individual and peer strengths, skills, and contributions
**Growth mindset**	Adolescents who embrace a growth mindset learn that they and their peers have the potential to develop their abilities.<br>*Discove*r uses growth mindset through:<br>• Introducing new concepts and activities that require increasing levels of difficulty to drive pursuit, persistence and mastery.<br>• Encouraging opportunities to learn from risk-taking and failure.<br>• Using activities that focus on the process of solving a problem and recognition of individual and peer improvement
**Curiosity**	Adolescence is a period of time where curiosity and experimentation are highly arousing and motivate learning. *Discover* gives opportunities for healthy exploration and risk-taking by:<br>• Giving opportunities for exploration to learn problems-solving<br>• Providing tools to seek out new information and knowledge<br>• Providing a safe space to practice taking individual and social risks to solve problems
**Purpose**	As adolescents are exploring their identity and values, exploring one's purpose can help develop long-term, heart-felt goals.

	Discover uses purpose in its activities by:<br>• Focusing on participant's strengths and values in activities<br>• Fostering gratitude for others and unique strengths<br>• Encouraging young people to reach out and learn from peers and trusted adults to pursue their goals
**Persistence**	Adolescence is a period of development that includes challenges and coping with rapid physical, social and emotional changes. *Discover* seeks to develop persistence by:<br>• Providing positive feedback and encouragement for effort<br>• Giving opportunities for young people to be involved in the planning process: identifying goals, planning the steps to achieve the goals, identifying potential obstacles and solutions<br>• Integrating increasing levels of difficulty in activities
**Generosity**	During adolescence there is increased tendency to explore social relationships outside of the family, including peer and community relationships. Generosity can help young people make connections, new friendships, and develop social support networks. *Discover* incorporates generosity by:<br>• Providing opportunities to celebrate victories in peer groups<br>• Encouraging perspective taking and conflict resolution skills.<br>• Encouraging intentional practice of gratitude and kindness
