# Supplementary material for: An Intervention to Enhance Social, Emotional, and Identity Learning for Very Young Adolescents and Support Gender Equity: Protocol for a Pragmatic Randomized Controlled Trial
Source: JMIR Res Protoc. 2020 Dec 31;9(12):e23071. doi: 10.2196/23071 (PMC7808886; doi:10.2196/23071)
Supplement: Multimedia Appendix 3 [file resprot_v9i12e23071_app3.pdf]

**Measurement Scale**	**Measurement Construct**	**Number of Questions Adapted**	**Cronbach's Measure of Reliability α**	**Response scale**
Simple Poverty Score Card, Tanzania	Household poverty	10	.26	Varies by question
Global Early Adolescent Survey	Sociodemographic & Adolescent health	10	.90	Varies by question
The Trait State Curiosity Scale	Curiosity	10	.84<br>.87	4-point Likert scale<br>*1 - strongly disagree to 4 - strongly agree*
The Dimensions of Identity Development Scale	Identity	16	.71<br>.72<br>.76<br>.90<br>.90	4-point Likert scale<br>*1 - strongly disagree to 4 - strongly agree*
The Scale for Measuring Persistence in Children	Persistence	10	.66	4-point Likert scale<br>*1 -strongly disagree to 4 - strongly agree*
The Interpersonal Generosity Scale	Generosity	10	.87	4-point Likert scale<br>*1 - strongly disagree to 4 - strongly agree*
The Young Lives Survey	Childhood poverty		.69<br>.80	Varies by question
The California Healthy Kids Survey (2017-18)	School engagement	10	.92	4-point Likert scale<br>*1 - strongly disagree to 4 - strongly agree*
Teamwork and Collaboration Assessment for High School	Teamwork and collaboration	13	.88<br>.80<br>.78	5-point scale<br>*1 - Never to 5 – Always*
Dweck Growth Mindset Questions	Growth mindset	9	.94 -.98<br>.85 -.94<br>.90 -.96	6-point scale<br>*1 - strongly agree to 6 - strongly disagree*
The Goal Orientation and Learning Strategies Survey	Goal orientation	14	.91 -.98	4-point Likert scale<br>*1 - strongly disagree to 4 - strongly agree*
The Global School-Based Students Health Survey	Bullying	7	.69<br>.90	Binary scale<br>*0 – no, 1 – yes*

The African Youth Psychological Assessment	Psychosocial adjustment	37	.72<br>.74<br>.88	4-point Likert scale<br>*1 - strongly disagree to 4 - strongly agree*
The Empathy Questionnaire for Children and Adolescents	Empathy	14	.70<br>.70<br>.74	3-point scale:<br>*1 - not true, 2 - sometimes, and 3 - often true*
The Gender Roles Equality and Transformations	Gender equality	13	.81	Binary responses<br>*1 – agree, 2 - disagree*
Self-Efficacy Questionnaire for Children	Self-efficacy	24	.85<br>.81<br>.65	5-point scale<br>*1 - not at all, 1 - a little bit, 2 - about average, 3 - well and 4 - very well*
Media and Technology Usage and Attitudes Scale	Technology use and uptake and attitudes	20	.61 - .97<br>.80 - .81	**Technology access:**<br>3-point scale: *1 - no, do not have, 2 - yes, but don't have my own and 3 - yes, I have my own*<br>**Technology usage:**<br>4-point scale: *1 - none, 2 - about 1 hour, 3 - about 2 hours and 4 - more than 2 hours*<br>**Technology perceptions:**<br>4-point scale: *1 - never, 2 - sometimes, 3 - often and 4 - always*
